# Rabies Post Exposure Vaccine Effectiveness: A Retrospective Case–Control Study in Amhara Region, Ethiopia

**DOI:** 10.1155/sci5/9307457

**Published:** 2026-02-20

**Authors:** Adane Bahiru, Sefinew A. Mekonnen, Liuel Yizengaw, Ambaye Kenubih, Wubneh Aklog, Yeshiwas Walle, Getahun Mihiret Chane, Abebe Tibebu, Teklu Yitbarek, Wudu T. Jemberu, Wassie Molla

**Affiliations:** ^1^ Amhara Agricultural Research Institute, Sekota Dryland Agricultural Research Center, P.O. Box 62, Sekota, Ethiopia, arari.gov.et; ^2^ Department of Veterinary Epidemiology and Public Health, College of Veterinary Medicine and Animal Sciences, University of Gondar, Gondar, Ethiopia, uog.edu.et; ^3^ Department of Veterinary Laboratory Technology, Debre Markos University, Debre Markos, Ethiopia, dmu.edu.et; ^4^ Department of Veterinary Pathobiology, College of Veterinary Medicine and Animal Sciences, University of Gondar, Gondar, Ethiopia, uog.edu.et; ^5^ Saint Peter Specialized Hospital, Addis Ababa, Ethiopia; ^6^ International Livestock Research Institute (ILRI), Addis Ababa, Ethiopia, ilri.org

**Keywords:** case–control, Ethiopia, post exposure vaccine, rabies, retrospective, vaccine effectiveness

## Abstract

Rabies can be prevented by vaccination of dogs and provision of post exposure vaccine (PEV) for exposed subjects. There are many post exposure rabies vaccines in the market with their efficacy extending to 100%; however, there are complaints on the effectiveness of the vaccine in the field level. Therefore, the aim of this study was to assess PEV effectiveness and identify factors associated with rabies cases. A retrospective case–control study was conducted in the Amhara regional state of Ethiopia from December 2020 to June 2021. Data were collected from a total of 138 subjects (92 controls and 46 cases). Descriptive statistics was used to summarize the data, and logistic regression model was used to identify risk factors. The vaccine effectiveness (VE) was determined by computing the percentage reduction in risk of rabies among vaccinated humans relative to unvaccinated individuals. Majority of rabies exposure (56.5%) were due to bite of stray dogs or dogs owned by others. About 65.2% of the study subjects had taken PEV. Subjects with rabies PEV were less likely to have rabies than subjects who did not take rabies PEV (OR = 0.058, 95% CI = 0.001–0.21), and the rabies PEV reduced 94.2% of the rabies cases. Females, children <15 years of age, and increased number of days from bite to PEV had higher odds of deaths due to rabies. There was a considerable reduction in the odds of rabies case in vaccinated humans as compared to that of nonvaccinated subjects. By focusing on target populations such as females and children under the age of 15 and by capitalizing health education on early search of PEV following dog bite, the reduction level rabies‐related death has the potential to be larger than the reported findings.

## 1. Introduction

Rabies is a viral disease associated with significant economic and public health impact. It is the reason for the death of 59,000 people, loss of 3.5 million disability adjusted life in years (DALYs), and about 8.6 billion USD [1]. The majority of deaths occur in Africa, where over 21,000 people die each year. Economic losses due to rabies are associated with premature death of humans and animals and costs incurred for management of the disease [2]. The largest portion of economic burden is associated with societal losses (premature death and seeking for post exposure prophylaxis (PEP) where only 1.5% of the expenditure is for dog vaccination service. Similarly, the largest share of economic losses is encountered in Asia and Africa [1].

In Ethiopia, reports indicate that rabies is considered as the top priority disease among zoonotic disease as reported by collective panels of experts from human, animal, and environmental scientists [3]. Additionally, Ethiopia is the second top country in Africa and fifth in the world where the largest number of death due to rabies is reported [1]. Nearly 3000 people develop and died of rabies, and 97,000 people seek for PEP costing the country on average 2 million USD annually [4]. However, the actual number of deaths is likely higher, as most deaths go unreported due to insufficient diagnostic facilities and monitoring systems. Given the quantity of recorded deaths, the possibility of underreported deaths, and the monetary losses, its public health importance is more serious and urgent.

Domestic dog bites are the predominant (99%) source of infection to humans [1]. The best way in eliminating rabies at the main source of infection to humans is vaccination of dog population [5]. For a better control of the disease and achieving herd immunity, it is reported that 70% of the dog population have to be vaccinated [6], however; a study in Addis Ababa, Ethiopia, indicates that only 26.86% of dogs were vaccinated against rabies [7]. The low coverage of dog vaccination and presence of large number of stray dogs alert that rabies seeks due attention. For wider vaccination coverage of dogs against rabies, creating population awareness about the importance of vaccination has to be addressed [8]. Although dog vaccination is key for elimination of the disease [9], its burden can also be minimized to a significant level through improved access of post exposure vaccination (PEV) and pre‐exposure prophylaxis (PrEP) [10, 11]. Therefore, the public health importance of the disease needs additional management options, PEV of exposed subjects.

The nerve tissue vaccine (NTV) and the cell culture vaccine are the rabies PEVs currently in use worldwide. Cell culture vaccines have been shown to have more immunogenicity, fewer side effects, and improved protection against rabies in both humans and animals. Nervous tissue vaccines, on the other hand, are known to result in systemic reactions including headache, nausea, and abdominal pain as well as local side effects like pain, redness, and swelling, which can lead to immune‐mediated neurological damage. Despite the World Health Organization’s recommendation to replace nervous tissue vaccines with safe and effective cell culture vaccines, they are still used throughout Asia and Africa; Ethiopia is no exception. Although NTV is less immunogenic than modern rabies cell culture vaccines, it has been used for a longer period of time in Ethiopia and has been shown to have the capacity to serve as postexposure prophylaxis when used appropriately. According to a study conducted in Ethiopia, roughly 29,610 doses are manufactured and distributed over the entire nation each year [12].

In Ethiopia, a locally produced 5% suspension of phenolized sheep brain tissue vaccine (NTV) is produced by the Ethiopian Public Health Institute (EPHI) and in use for the control rabies for exposed subjects [13]. Then, the EPHI distributes the vaccine to regional admirations in accordance with the demand they had requested, though there is a limitation in providing the required volume due to limited production capacity. The regional governments subsequently placed in health facilities that are deemed to be equally accessible to the whole public. Despite efforts to make the vaccines available, they are concentrated in limited places where millions of people live. In Amhara region, there are about seven central health institutions providing the PEV covering an area of 250,709 km^2^ and serve over 24 million people when the study was conducted. The community may experience a delay in receiving the vaccine due to these distant access points, which could lead to issues with its effectiveness. The post exposure vaccine, currently in the market, is effective with efficacy extending up to 100% [14]. Moreover, there are complaints associated with nervous tissue vaccine on its route of administration, availability, cost, immunogenicity, and vaccine‐related complication [12]. Due to these reasons, significant portions of the population rely on plant remedies that are not scientifically approved for their efficacy [15]. Additionally, there is a delay in getting a PEV, which should happen right away after being bitten by a dog.

Vaccine efficacy trials are being conducted from producing companies before it is prescribed to the wider population. In addition to clinical efficacy trials, this demands rabies vaccine effectiveness studies that reveal the level of protection in the field and help for better policy recommendations [16]. Observational (case–control) study on vaccine effectiveness is known to indicate the real performance of the vaccine on the population where efficacy trial is limited in measuring the potency of the vaccine in a controlled environment [17]. Thus, even with the availability of an effective vaccine, it is good to consider vaccine effectiveness study from retrospective data as an extension to the vaccine efficacy done in making decisions since effectiveness is better in reflecting the real‐world situation where the vaccine and vaccination service are there [18]. Therefore, the presence of limited options for modern PEVs, a lack of dog vaccination customs and services, and complaints about the NTV, it is necessary to accurately assess the vaccine’s efficacy at the population level. Therefore, the aim of this case–control study was to assess PEV effectiveness of nervous tissue vaccine and identify factors associated with rabies cases in Amhara region, Ethiopia.

## 2. Methods

### 2.1. Study Area and Population

The study was conducted in the Amhara regional state of Ethiopia. The region is located in Northern and Northwestern part of the country that is composed of 12 administrative zones. The region has a diverse agroecological pattern and can be aggregated into high land to extreme high land, mid‐land, and low land with elevation ranging from 600 to 4600 m above the sea level [19]. The study populations were subjects with rabies death (case) and recovery (control) following a rabid dog bite.

### 2.2. Study Design and Operational Definition

A retrospective case–control study was used to evaluate rabies post exposure vaccine effectiveness in humans. Retrospective data of two years (December 2018 to November 2020) were collected for cases and controls by interviewing bitted victims, or parents of bitted victim’s family. The whole data collection was done from December 2020 to June 2021.

#### 2.2.1. Cases

Cases were subjects who had a known rabid dog bite and died due to rabies. Subjects were considered as cases based on the overt clinical signs (such as acute neurological syndrome (encephalitis) dominated by forms of hyperactivity followed by paralytic syndromes that progresses toward coma and death, usually by respiratory failure, within 4–7 days after the neurologic signs were observed) they had exhibited and died following bite with dogs suspected of having rabies regardless of their PEV status. Additionally, care was taken to avoid the inclusion of deaths due to other reasons by considering the history of subjects before bite.

#### 2.2.2. Controls

Controls were subjects who had a known bite of rabies‐suspected dog but did not die from rabies and recruited from the same area (village or the nearest residence) where dead humans (cases) due to rabies were enrolled in the study. These controls were people who had survived dog bites that were thought to be the cause of human deaths (cases) regardless of their PEV status.

#### 2.2.3. Post‐Exposure Vaccine (PEV) Effectiveness

PEV effectiveness is the measure of the percent of reduction of rabies cases contributed by the protection effect of rabies PEV in the real‐world condition than limited in measuring the potency of the vaccine in a controlled environment. Opposed to vaccine efficacy studies, subjects in vaccine effectiveness are selected based on predecided exposure history than through proper randomization and allocation of subjects into cases and controls in the experiment [20]. Additionally, PEV effectiveness does not indicate inherent efficacy of the vaccine; rather, it reflects the level of reduction of deaths due to the variability of various factors and system of administration.

#### 2.2.4. Vaccine Used, Schedule, and Full Course Considered as Vaccinated in the Study

Analysis of its effectiveness was based on exposure history from a locally produced 5% suspension of phenolized sheep brain tissue. The entire course of the vaccination was administered by subcutaneous injections around the umbilicus for 14 days in a row, followed by three booster doses with a volume of 2–5 mL on the 10th, 20th, and 30th days of the last injection as recommended by the Ministry of Health and EPHI [21]. In most of the cases and service provider health centers, nervous tissue vaccines are the only choices in use. Other modern post exposure vaccines and immunoglobulin are not incorporated in the study as there are no enough data to draw conclusion on it; basically it is rare to have these management options in most of the service providing health centers. History of immediate wound wash and rinsing and status related to rabies immune globulin (RIG) provision were not considered as a component of PEV.

#### 2.2.5. Data Collection and Recruitment of Cases and Controls

Dog bite and rabies situation were our interest for the vaccine effectiveness study. Data for both history of rabies situation and exposure to rabid dog bite were collected by interviewing family members in person and from hospital and clinic public health surveillance records. As there was no formal recording of rabies situation and exposure of rabid dog bite, we tried to validate the data by triangulating the response with more than one individual when a respondent was suspected doubtful. During the data collection, the date at which the dog bite accident was occurred, date of vaccination provided, ownership of the biting dog, the date of death of person, and symptoms defining rabies were recorded. Moreover, sociodemographic data of respondents were collected. Subjects who had known bite from a suspected rabid dog (recovered or died) and their PEV status (vaccinated or nonvaccinated) were recorded. Those individuals who had received any rabies vaccination at any point after exposure to suspected rabid dog bite and had completed the full course of the vaccine were included for the evaluation of its effectiveness as a vaccinated group. However, those subjects who failed to complete the full course of the vaccine were considered as “No PEV” and they were considered as nonvaccinated.

Rabies clinical case definition was developed to better classify dogs as positive or negative for rabies through clinical diagnosis. A case definition of hypersalivation, paralysis, lethargy, unprovoked abnormal aggression, abnormal behavior followed by paralysis and death was used for the classification of dog as positive or negative for rabies, and this was assessed in two ways. The first was if dog was under follow‐up and develops signs of paralysis following furious stages and the dog ends with death, it was considered as rabid and any bite by this dog was considered as exposure to rabies. The second approach when the biting dog was not under follow‐up after biting (stray dogs or those immediately killed by owners). In this case, when the assessment was based on the outcome on the bitten subject (cases) if the outcome for the bitten subjects was death with typical signs of rabies, the biting dog is considered as rabid.

### 2.3. Sample Size Determination

Sample size required for this study was determined based on previous reports by Jemberu et al., [15] who reported that from humans and dogs bitten by a rabid dog, 29% developed rabies and end up with death. A report in Hayti, which is indicated to be applicable in low‐income countries of Africa and Asia, indicated that an integrated bite case management with vaccination can reduce the death of human by 65% [22]. Therefore, the death of humans with the application of PEP was expected to be 35% (65% of the cases could be reduced through integrated management) of 29% (10.15%). Although the use of equal number of cases and controls is possible, we used a double count of the number of controls over the cases to enhance statistical power [23]; due to predetermined nature of the data collection, matching between cases and controls was not done. The sample size required was calculated using 90% confidence interval and 80% power in Epi_info_7 statistical software following the Kelsey sample size estimation ratio [24]. Based on the calculation, a total of 138 subjects (92 controls and 46 cases) were used for the vaccine effectiveness and risk factor identification study.

### 2.4. Study Variables

**FIGURE 1:**
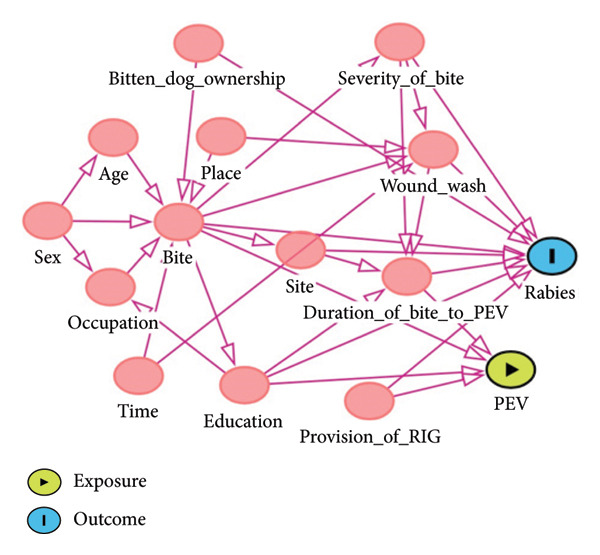
A causal diagram of factors affecting rabies case occurrence in humans.

Variables deemed potential risk factors for rabies case were included in the questionnaire, and their causal diagram of measured and unmeasured variables were considered. It is clear that not all dog bites are linked to rabies infections. However, there are other circumstances in which a dog’s typical behavior is altered, and it is crucial to rule out behavioral changes brought on by infection. Dogs have a tendency to be aggressive at night, in the morning, and in the evening, which may be related to their desire for food, walks, and fear. When other dogs approach their area, they become hostile and have a tendency to bite both humans and other dogs. This is another aspect contributing to their aggressiveness. Some people must visit households because of their line of work, which increases the likelihood that they will come into contact with potentially rabid canines. The potential risk factors are indicated in the causal diagram (Figure 1). Each site of infection and occupation were classified into five levels, each of time of bite and age were classified into three levels, and the rest of the variables were classified into two levels. The level of severity of bite wounds was classified into three categories. Category 1: touching or feeding animals, animal licks on intact skin (no exposure); category 2: nibbling of uncovered skin, minor scratches or abrasions without bleeding (exposure); and category 3: single or multiple transdermal bites or scratches, contamination of mucous membrane or broken skin with saliva from animal licks, and exposures due to direct contact with bats (severe exposure) as described by World Health Organization. Bites of category 2 and category 3 were considered as exposure to rabid dog bite [25] and included in this study.

### 2.5. Data Management and Statistical Analysis

The data were recorded in excel and checked for completeness and exported to version 16 of the STATA software package. Descriptive statistics such as percentiles and frequencies were used to summarize demographic characteristics of respondents and distribution of cases and controls.

Logistic regression models were used to test the association between sociodemographic variables and rabies case. Analysis of vaccine effectiveness was done by comparing cases and controls in the vaccinated and nonvaccinated groups. Independent variables statistically significant with *p* value < 0.25 were further tested in multivariable logistic regression models. The model equation is presented in (1). *p* *P* value < 0.05 was considered statistically significant association in the multivariable analysis. Odds ratio and 95% CI were also calculated. The vaccine effectiveness was calculated by using adjusted odds ratio with the formula: VE = (1 − adjusted odds ratio for PE vaccination status) ∗ 100% [26].

(1) LogitPi=β0+β1X1+β2X2+⋯+βnXn+Є,

where *P*_*i*_ = the likelihood of rabies exposed subject to have rabies case and end up with death, *β*_0_ = the constant, *β*_1_, *β*_2_, … *β*_*n*_ = the regression coefficients, *X*_1_, *X*_2_,…, *X*_*n*_ = PE vaccination status and other predictor variables, and *Є* = the residual error.

A multicollinearity test was done before fitting the variables into multivariable logistic regression model to rule out a significant correlation between predictor variables. If the value of variance inflation factors (VIFs) was lower than 10, then the collinearity problem was less likely [27]. Confounding and interactions were checked for variables in the final model. The backward stepwise elimination procedure was used to fit the variables while checking for confounding, which was considered present if the remaining coefficients changed at least 25% after removing a nonsignificant (*p* > 0.05) variable from the model. Interactions were tested for all combinations of the significant main effects in the final multivariable logistic regression.

### 2.6. Ethical Approval

In accordance with college guidelines for ethics, questionnaire studies involving humans without any intervention are not required to obtain ethical approval, and only verbal agreement with parents of participants less than 18 years of age is necessary. The Ethical Clearance Committee of the College of Veterinary Medicine and Animal Sciences (CVMAS, Ethical clearance Committee ref. no. 1065/2022), University of Gondar, Gondar, Ethiopia, approved the study based on the ethical guidelines. In addition to the declared ethical approval, we got the study participants’ informed verbal agreement and the parents’ signed consent (for individuals under the age of 18).

## 3. Results

### 3.1. Descriptive Statistics

Majority of the study subjects (70%) were males and around 51% were within the age range of 15–45 years. Large portion of the individuals with a rabid dog bite (65.2%) had taken the full course of PEV against rabies. Most of the cases were individuals who were bitten on their legs (54.3%) and from wound severity level of category 3 (89.1%). Almost half of the bites (70) were recorded in the morning time (Table 1).

**TABLE 1:** Background characteristics of 138 individuals with a history of rabid dog bite.

Factors	Category	Rabies status		Total *N* (%)
		Number of cases (%)	Number of controls (%)	
Sex	Female	20 (43.5)	21 (22.8)	41 (29.71)
	Male	26 (56.5)	71 (77.2)	97 (70.29)

Age	< 15	19 (41.3)	23 (25.0)	42 (30.43)
	15–45	16 (34.8)	54 (58.7)	70 (50.72)
	> 45	11 (23.9)	15 (16.3)	26 (18.84)

Education	Illiterate/informal learning	21 (45.7)	24 (26.1)	45 (32.6)
	Formal learning	25 (54.3)	68 (73.9)	93 (67.4)

Occupation	Farmer	16 (34.8)	32 (34.8)	48 (34.78)
	Student	19 (42.2)	38 (41.3)	58 (42.03)
	Civil servant	2 (4.4)	10 (10.9)	12 (8.70)
	Merchant	2 (4.4)	4 (4.3)	6 (4.35)
	Child (under age)	6 (13.3)	8 (8.7)	14 (10.14)

PE vaccination status	Vaccinated	16 (34.8)	74 (82.2)	90 (65.2)
	Nonvaccinated	30 (65.2)	18 (19.6)	48 (34.8)

Ownership of the biting dog	Nonown or stray dog	26 (56.5)	52 (56.5)	78 (56.5)
	Own dog	20 (43.5)	40 (43.5)	60 (43.5)

Site of exposure	Thigh	10 (21.7)	16 (17.4)	26 (18.84)
	Leg	25 (54.3)	45 (48.9)	70 (50.72)
	Palm	1 (2.2)	7 (7.6)	8 (5.80)
	Belly	2 (4.3)	6 (6.5)	8 (5.80)
	Arm	8 (17.4)	18 (19.6)	26 (18.84)

Place of bite	Village	26 (56.5)	52 (56.5)	78 (56.52)
	Homestead	20 (43.5)	40 (43.5)	60 (43.48)

Severity of bite	Category 2	5 (10.9)	51 (55.4)	56 (40.6)
	Category 3	41 (89.1)	41 (44.6)	82 (59.4)

Time of bite	Day time	21 (45.7)	35 (38.0)	56 (40.58)
	Early morning	20 (43.5)	50 (54.3)	70 (50.72)
	Evening/night	5 (10.9)	7 (7.6)	12 (8.70)

### 3.2. Factors Associated With Rabies Cases

The multivariable logistic regression analyses had shown that six variables were significantly associated with rabies. The post exposure vaccine effectiveness in the current system of delivery is 94.2% (VE = (1 − odds ratio of rabies status between vaccinated and nonvaccinated) ∗ 100%). The likelihood of occurrence of rabies case was associated with the duration from bite to receiving post exposure vaccine, indicating the higher likelihood of occurrence of rabies death for each day tardiness in taking PEP (OR = 1.15, 95% CI = 1.05–1.26) (Table 2).

**TABLE 2:** Summary of the models describing univariable (*p* < 0.25) and multivariable (*p* < 0.05) associations between independent variables and rabies based on data from 138 individuals with suspected rabid dog bite in Amhara region, Ethiopia.

Variables	Category	COR (75% CI)	*p* value	AOR (95% CI)	*p* value
PE vaccination status	Nonvaccinated	Ref			
	Vaccinated	0.13 (0.059–0.288)	0.001^∗^	0.058 (0.001–0.21)	0.001^∗^

Severity of bite	Category 2	Ref			
	Category 3	10.20 (3.69–28.16)	0.001^∗^	13.44 (2.28–79.15)	0.004^∗^

Site of bite	Thigh	Ref			
	Leg	0.89 (0.35–2.25)	0.804		
	Palm	0.23 (0.02–2.15)	0.196		
	Belly	0.53 (0.09–3.18)	0.490		
	Arm	0.71 (0.23–2.24)	0.561		

Occupation	Farmer	Ref			
	Student	1.05 (0.47–2.36)	0.901		
	Civil servant	0.40 (0.08–2.05)	0.271		
	Merchant	1.00 (0.17–6.05)	0.100		
	Child (under age)	1.50 (0.44–5.06)	0.51		

Sex	Female	Ref			
	Male	0.39 (0.18–0.82)	0.014^∗^	0.07 (0.01–0.37)	0.003^∗^

Age	< 15	Ref			
	15–45	0.36 (0.16–0.82)	0.015^∗^	0.25 (0.04–0.86)	0.031^∗^
	> 45	0.89 (0.33–2.38)	0.813	2.69 (0.38–19.34)	0.324

Education	Illiterate/informal learning	Ref			
	Formal learning	0.42 (0.19–0.88)	0.02	0.37 (0.13–0.77)	0.04

Place of bite	Village	Ref			
	Homestead	1.00 (0.48–2.04)	1.00		

Ownership of the biting dog	Nonown or stray dog	Ref			
	Own dog	1.00 (0.48–2.04)	1.00		

Time of bite	Day time	Ref			
	Early morning	0.66 (0.31–1.41)	0.28		
	Evening/night	1.19 (0.33–4.23)	0.78		

Duration in days	Continuous variable	1.14 (1.06–1.22)	0.001^∗^	1.15 (1.05–1.26)	0.002^∗^

*Note:* Ref = reference category.

Abbreviations: AOR = adjusted odds ratio, COR = crude odds ratio.

^∗^Indicates a significant difference at *p* value of 0.05.

## 4. Discussion

The objectives of this study were to understand PEV effectiveness and identify factors associated with rabies cases specific to the situation in Amhara region, Ethiopia.

The results of this case–control study indicated that the effectiveness of the vaccine is found to be 94.2%. Vaccines recommended by the World Health Organization and currently in use can reduce the expected number of rabies cases to extremely low [28] and prevent 56,000 deaths annually [27]. Proper handling, wound management, timely application, and uptake full dose of the vaccine were recommended to enhance its effectiveness [29]. Even while the vaccination lowers the death rate of exposed individuals, the observed field‐level death in vaccinated population is unacceptable because there is a potential that the vaccine can lower the death rate to extremely low levels. The study shows that though the disease is avoidable, it is now fatal in the region. This paper discusses factors that may be important for future control and prevention of the disease, responsible for decreased vaccine effectiveness and the incidence of rabies‐related mortality. Failure to take the whole doses, a delay in search for post exposure vaccine and a big rely on traditional medicines from plant remedies, and consequently search for post exposure vaccine after the clinical signs developed were among the reason for the observed rabies cases with the application of PEV.

Developing rabies symptoms (being rabies case) varied depending on the severity of the bite; bites with category 3 developed to rabies more than bites with wound category 2. The result is in line with established knowledge that wound severity affects the transmission probability and the amount of the virus to be inoculated [30]; thus, the observed difference is expected. Though rabies immunoglobulin is recommended to be used as an important component of PEP, especially in exposure with severe wound involvement, it is rarely used in the regional rabies preventive package because of issues with availability and application costs, and it is not taken into consideration here. The majority of bites (56.5%) were by stray dogs. This finding is in line with the report of Gebru et al. [31] in Northwestern Tigray who found 80.4% rabies exposures caused by stray dog bites. This finding indicates that stray dogs have a greater tendency to be infected by rabies while moving freely without restriction and transmit the disease than owned dogs or their number might be larger than owned dogs and increase the infection pressure of rabies compared to owned dogs. However, we found that owned dogs also caused a considerable share (43.5%) of rabies exposure. The current result is higher than previous work of Abubakar and Bakari [14] from Nigeria who reported that only 14% bite injuries for rabies exposure were by own dogs. It is also a contradictory with previous statements where dog ownership was mentioned as a major factor capable of predisposing dogs to rabies where owned dogs access to rabies preventive measures and low exposure pressure is expected [32]. Whereas in this study a considerable number of rabies cases are encountered from dogs with ownership, this might be associated with low management care (vaccination, restriction of movement) of owners for their pets. A high number of stray dogs, a failed vaccination schedule, and a lack of public knowledge of the need to vaccinate dogs are the main causes of the high infection pressure from both owned and stray dogs. Most of the time, the community kills biting dogs when it would be better to study the dog’s clinical state if it were allowed to be confined instead of killing it. This is an additional challenge for medical practitioners who must deliver postexposure immunizations for any dog bites that might not have been infected. Given the extreme lack of vaccinations, this presents a challenge to long‐term disease control and lowering human mortality.

The final multivariable logistic regression model analysis shows vaccination, duration in days from time of bite to the time taking post exposure vaccine, educational status, severity of bite, age, and sex of the individuals are important factors associated with cases of rabies. Females were more likely to develop rabies than males. The higher likelihood of rabies cases in females contradicts a previous report by Deressa et al. [33]. This might have been related to the relatively higher number of rabies exposures occurred from own dogs (43.5%) and the nature of work responsibilities of females in Ethiopia; females are mostly responsible for activities in and around the home such as cooking, child care, and other activities in homestead that increased the chance of contact to own dogs. A study in Amhara region, Ethiopia, had also reported that females were more likely to develop rabies than males because they had low knowledge about transmission and preventive measures of the disease [34]. Reports indicate that males have higher access to information related with rabies prevention and control from health extension agents and have a better chance of practicing preventive measures [35]. This would not be surprising as most of extension contacts especially in rural parts of the country are biased toward males.

The severity of the wound, where bites with category‐III were more likely to develop rabies than those with category‐II, is another significant element that affects rabies. This is to be expected since severe bites increase the risk of infection, increase the likelihood that rabies will occur, and ultimately result in death. Furthermore, compared to those who are illiterate, those with formal education have a comparatively lower risk of contracting rabies. This is also not surprising because educated people are more likely than illiterate persons to have access to preventative interventions [34].

Subjects with age group of 15–45 years had lower odds of rabies than groups less than 15 years of age. This finding is in agreement with previous works who reported that children were more likely to be exposed to rabies than adults in Ethiopia [31, 33] and other countries [36, 37]. Unlike our findings, Zeynalova et al. [38] from Azerbaijan indicated that adults were more commonly affected than children. The differences in the findings of rabies cases among age groups could be attributed to sociocultural differences among various communities. For instance, in one type of community, adults could be at a higher risk of rabies due to the fact that they usually conduct outdoor activities at distant places away from home. In another community, children might be more commonly exposed to rabies because they are not well attended. Besides, children have always a tendency to play with dogs and have low awareness as well as they frequently chase and/or throw stones, which can provoke dogs. Another reason could be that women are more likely than men to be involved in child care, and female participants were less likely than male participants to have adequate knowledge preventive practices [34] that could affect the children probability of having PEV as early as possible.

Even with the application of vaccination a day, delay in taking PEV increased the likelihood of the subject to develop clinical disease and death by 1.15. This supports our knowledge of the need for taking PEP as quick as possible following a bite [10]. Similar findings have been reported in Ethiopia; a delay in taking PEV for more than 3 days has been seen and maximizes the probability of infection and subsequently death [39]. One reason for the delay of taking PEV could be poor accessibility of rabies PEV and the search for traditional healers. Related to this, it was reported that residents within the study area believe that traditional healers can diagnose the disease [15] and the quick search for PEV might be affected. Up on our observation, there is a big reliance with plant remedies, which are not scientifically proven for their effectiveness and are thought to be more effective in early cases than nervous tissue vaccines. Another problem with finding PEV as soon as possible is accessibility; the region’s inadequate infrastructure and the existence of centralized medical health centers make it challenging to get vaccination the earlier possible.

### 4.1. Limitations of the Study

The classification of humans and dogs into positive or negative for rabies was based on only clinical diagnosis using clinical case definition of rabies. Some misclassification may be occurred as we did not use confirmatory tests such as fluorescent antibody test (FAT) and reverse transcription polymerase chain reaction (RT‐PCR). This can be considered as a limitation of this study. However, as rabies has obvious symptoms and invariably fatal consequence, the misclassification would not be that much. The other limitation of the study was that potential confounders of rabies occurrence such as a history of immediate wound wash and rinsing and status related to RIG provision were not considered and controlled in the study. Because it is difficult to define wound wash consistently, which could further confound the assessment, it is not considered as a factor in the development of rabies. Another drawback is that people with partial PEV are not regarded as confounders because the incompleteness cannot be consistently defined to be taken into account in the analysis.

## 5. Conclusion

In general, the majority of rabies management alternatives do not adhere to the WHO criteria for using PEV against exposed individuals. The largest share of the people do not take into account the requirement for early wound care, follow‐ups with the biting dog, and the prompt search for postexposure immunization after a dog bite. Additionally, even though the WHO prohibits the use of nerve tissue vaccinations, they are essentially the only vaccine available in service provider health centers, and the effectiveness we observed is fairly good when used in accordance with standard use guidelines. There was a considerable reduction in the odds of rabies case and death in vaccinated than unvaccinated population. Even though the field level effectiveness is lower than the efficacy in clinical trial, a significantly high proportion of the population death due to rabies is reduced by the application of PEV. Female and children under 15 years of age, those who did not attend formal school learning, and a delay to take PEV are significantly associated with rabies positivity and death. We suggested that health extension service should focus on educating females and giving attention to subjects younger than 15 years of age. Public awareness on taking rabies PEV as quick as possible following dog bite needs to be promoted.

## Author Contributions

Conceptualization: Adane Bahiru, Wassie Molla, Wudu T. Jemberu, and Sefinew A. Mekonnen.

Data curation: Adane Bahiru, Liuel Yizengaw, Ambaye Kenubih, Wubneh Aklog, Yeshiwas Walle, Getahun Mihiret Chane, Abebe Tibebu, and Teklu Yitbarek.

Formal analysis: Adane Bahiru, Wassie Molla, and Sefinew A. Mekonnen.

Investigation: Wassie Molla, Wudu T. Jemberu, and Sefinew A. Mekonnen.

Methodology: Adane Bahiru, Wassie Molla, Wudu T. Jemberu, and Sefinew A. Mekonnen.

Supervision: Wassie Molla, Wudu T. Jemberu, and Sefinew A. Mekonnen.

Validation: Wassie Molla, Wudu T. Jemberu, and Sefinew A. Mekonnen.

Writing–original draft: Adane Bahiru.

Writing–review and editing: Adane Bahiru, Sefinew A. Mekonnen, Liuel Yizengaw, Ambaye Kenubih, Wubneh Aklog, Yeshiwas Walle, Getahun Mihiret Chane, Abebe Tibebu, Teklu Yitbarek, Wudu T. Jemberu, and Wassie Molla.

## Funding

No funding was received for this manuscript.

## Conflicts of Interest

The authors declare no conflicts of interest.

## Data Availability

The data that support the findings of this study are available from the corresponding author upon reasonable request.
